# Supplementary material for: Multiple rotavirus species encode fusion-associated small transmembrane (FAST) proteins with cell type-specific activity
Source: J Virol. 2025 Nov 12;99(12):e01587-25. doi: 10.1128/jvi.01587-25 (PMC12724187; doi:10.1128/jvi.01587-25)
Supplement: Supplemental figures — Figures S1 to S8. [file jvi.01587-25-s0001.pdf]

SUPPLEMENTARY MATERIAL

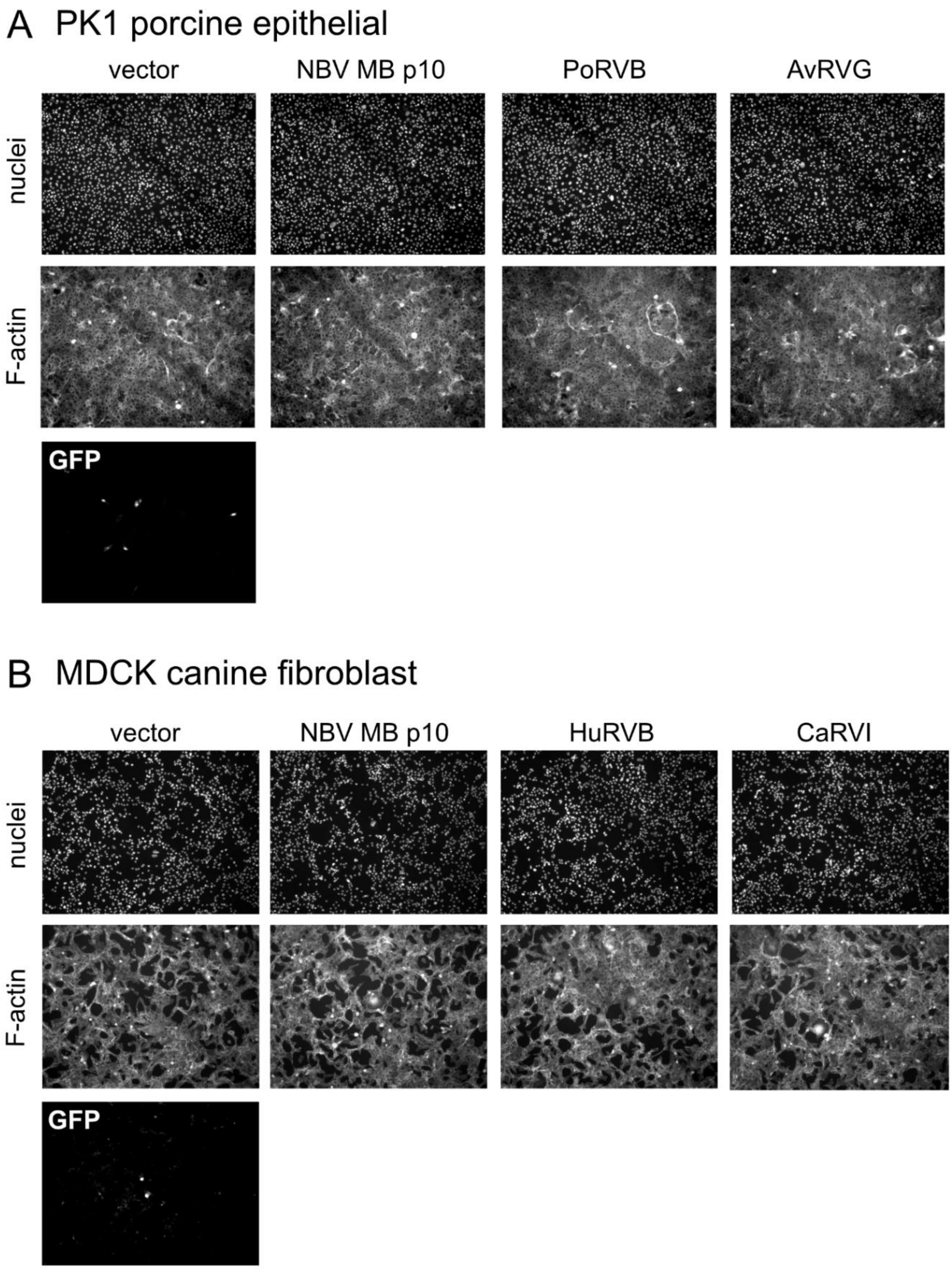

Figure S1. RVB, RVG, and RVI NSP1-1 fail to form syncytia in pig and dog cells. DAPI-stained (nuclei) and rhodamine phalloidin-stained (F-actin) images of porcine PK1 (A) and canine MDCK (B) cells transfected with vector, NBV p10, GFP, and RVB, RVG, or RVI NSP1-1. Images for selected constructs are shown to represent negative (vector) and potential positive (NBV p10 in DF-1 cells) controls and the level of transfection (GFP), as well as for selected NSP1-1 proteins.

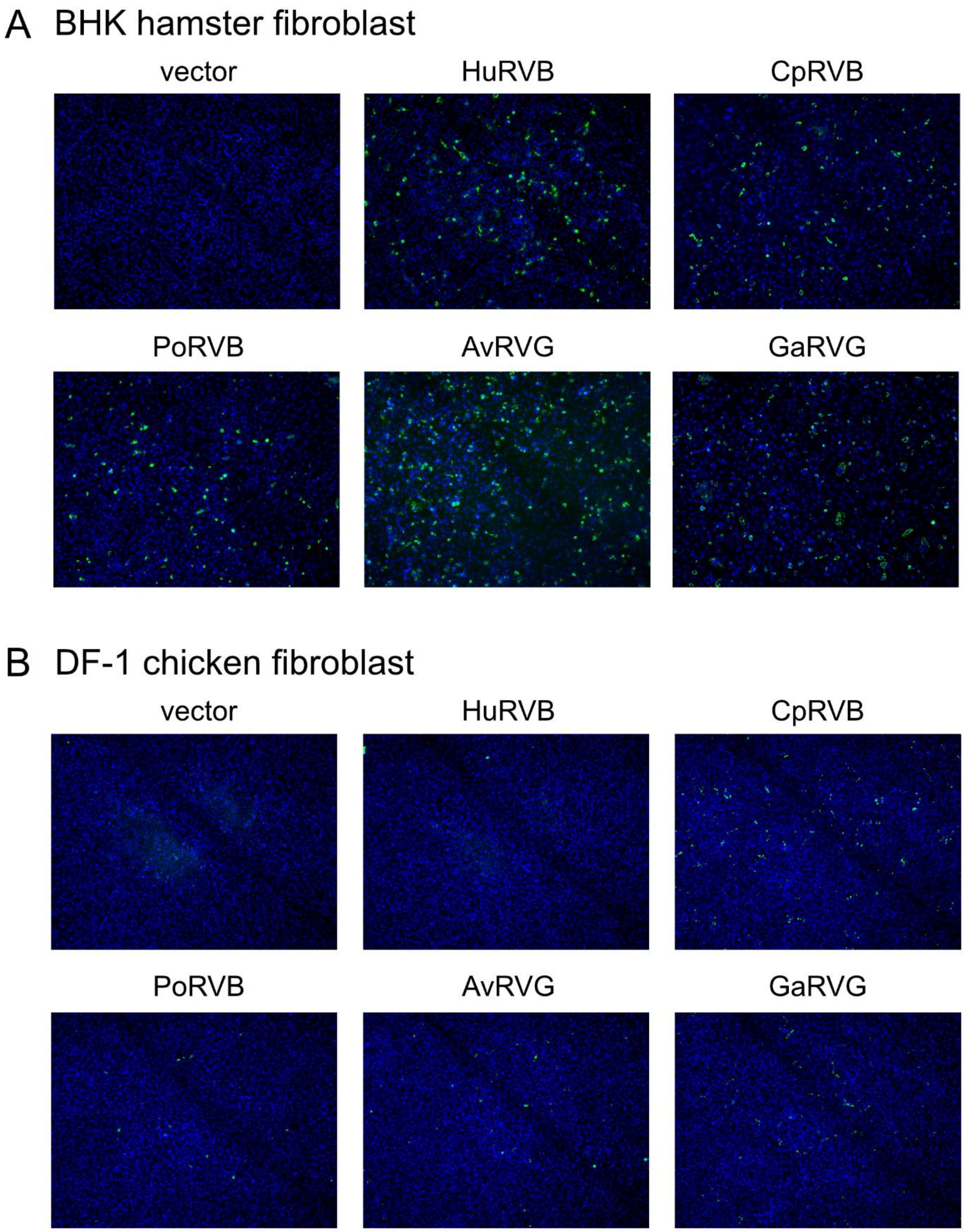

Figure S2. Detection of nuclei and NSP1-1 protein in hamster and chicken cells transfected with plasmids expressing NSP1-1-FLAG. Hamster BHK fibroblasts (A) or chicken DF-1 fibroblasts (B) were transfected with pCAGGS alone (vector) or pCAGGS expressing the indicated C-terminally FLAG-tagged NSP1-1 protein. After 18h, transfected cells were stained to detect nuclei (blue, DAPI) and FLAG peptide (green).

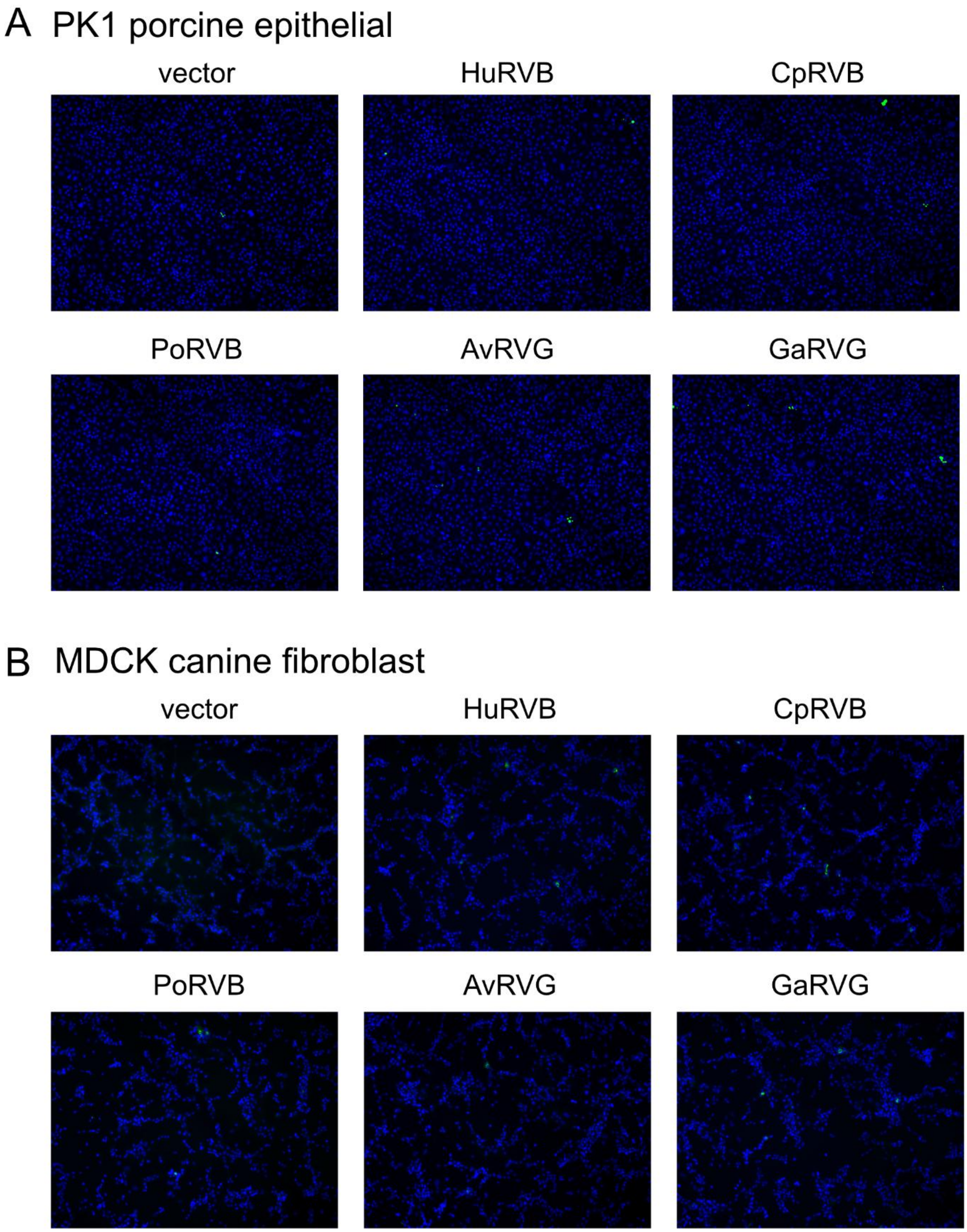

Figure S3. Detection of nuclei and NSP1-1 protein in pig and dog cells transfected with plasmids expressing NSP1-1-FLAG. Porcine PK1 epithelial cells (A) or canine MDCK fibroblasts (B) were transfected with pCAGGS alone (vector) or pCAGGS expressing the indicated C-terminally FLAG-tagged NSP1-1 protein. After 18h, transfected cells were stained to detect nuclei (blue, DAPI) and FLAG peptide (green).

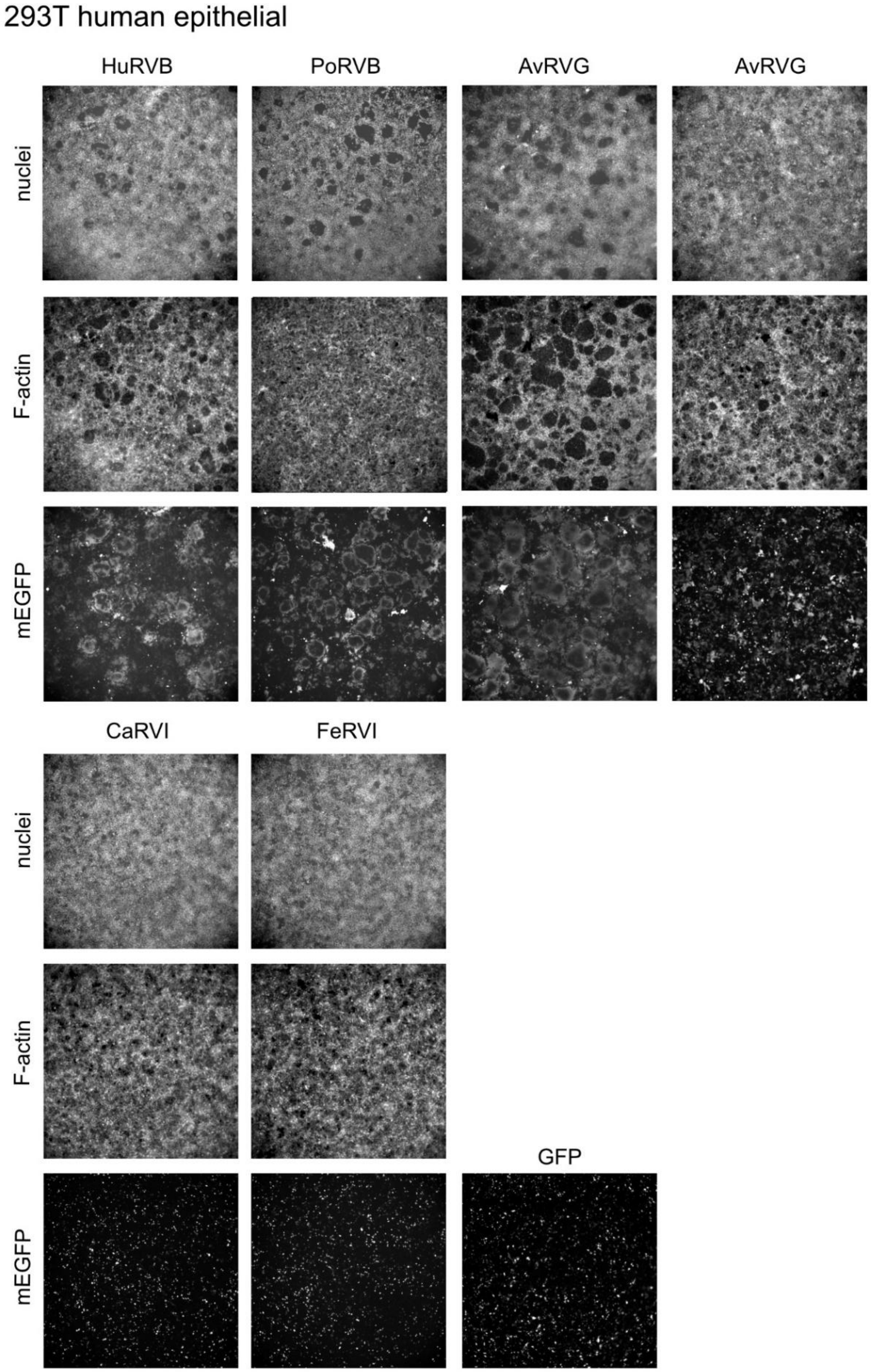

Figure S4. Detection of nuclei, actin, and mEGFP in 293T human kidney epithelial cells transfected with plasmids expressing a bicistronic RNA. 293T cells were transfected with plasmids encoding RVB, RVG, or RVI NSP1-1 and mEGFP separated by an IRES on a bicistronic RNA or GFP alone. Transfected cells were stained to detect nuclei (DAPI) and F-actin (rhodamine phalloidin).

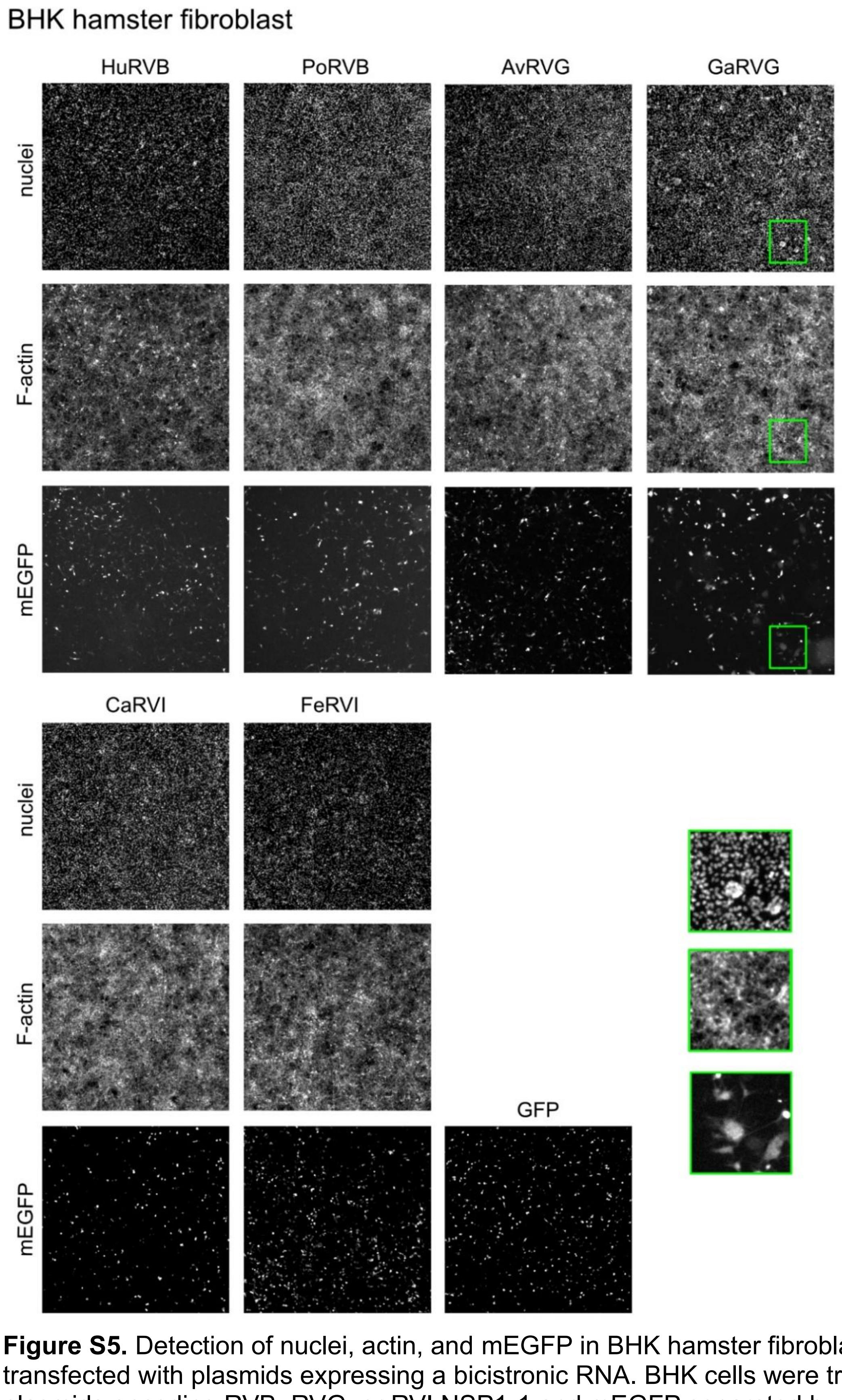

Figure S5. Detection of nuclei, actin, and mEGFP in BHK hamster fibroblasts transfected with plasmids expressing a bicistronic RNA. BHK cells were transfected with plasmids encoding RVB, RVG, or RVI NSP1-1 and mEGFP separated by an IRES on a bicistronic RNA or GFP alone. Transfected cells were stained to detect nuclei (DAPI) and F-actin (rhodamine phalloidin). Digitally cropped and magnified insets showing syncytia from the GaRVG images are shown to the right of the GFP image.

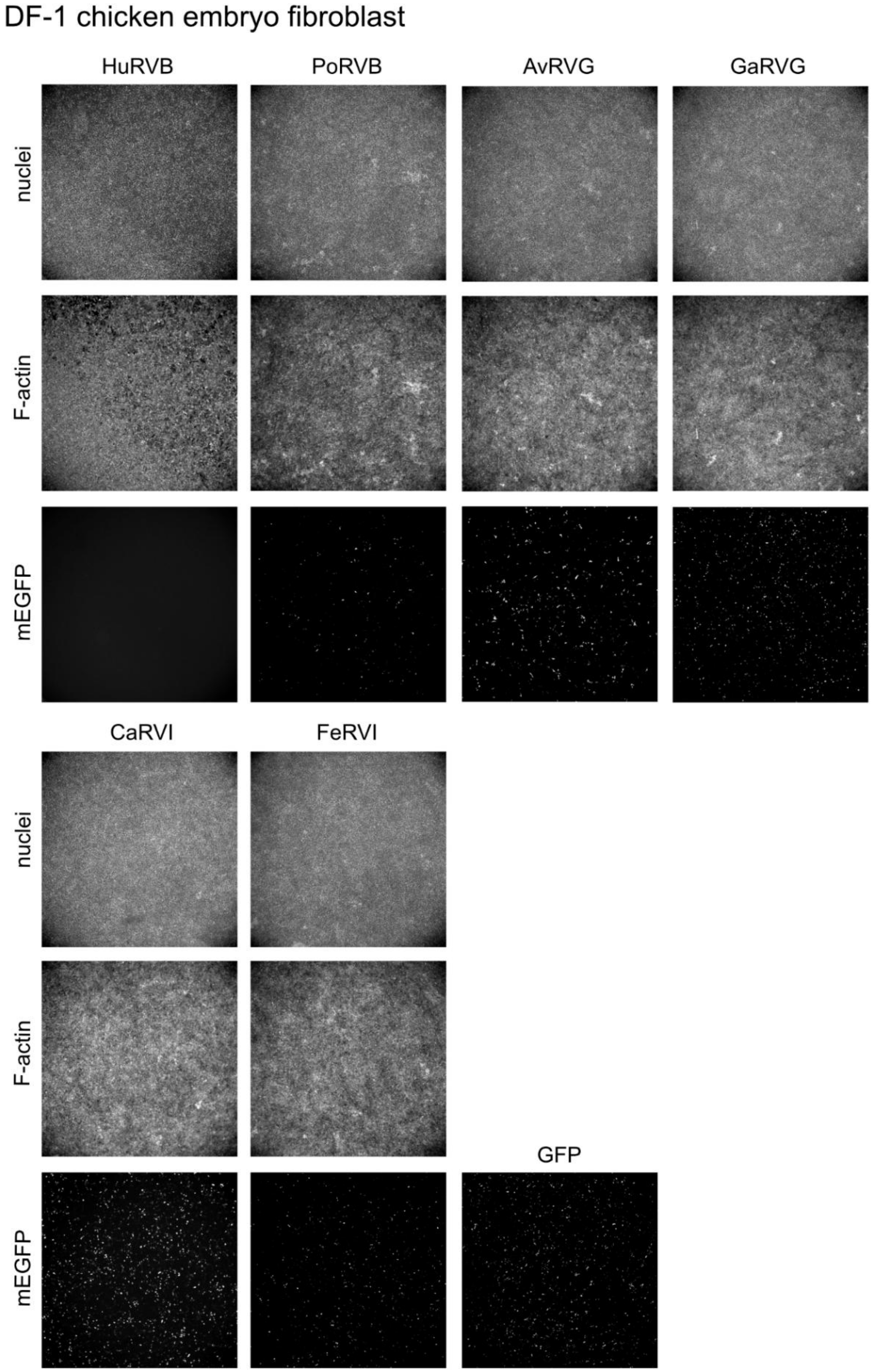

Figure S6. Detection of nuclei, actin, and mEGFP in DF-1 chicken embryo fibroblasts transfected with plasmids expressing a bicistronic RNA. DF-1 cells were transfected with plasmids encoding RVB, RVG, or RVI NSP1-1 and mEGFP separated by an IRES on a bicistronic RNA or GFP alone. Transfected cells were stained to detect nuclei (DAPI) and F-actin (rhodamine phalloidin).

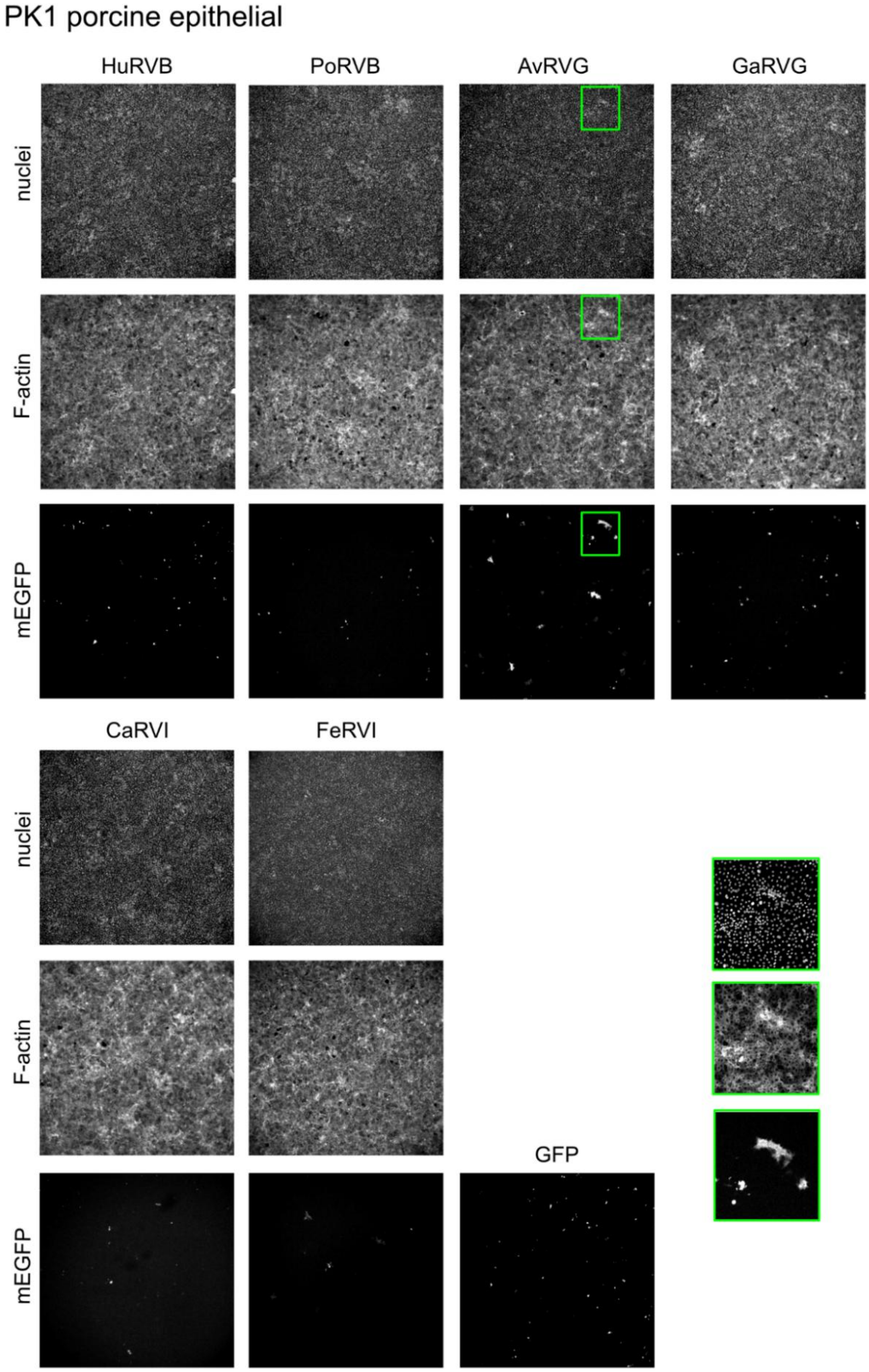

Figure S7. Detection of nuclei, actin, and mEGFP in PK1 porcine epithelial cells transfected with plasmids expressing a bicistronic RNA. PK1 cells were transfected with plasmids encoding RVB, RVG, or RVI NSP1-1 and mEGFP separated by an IRES on a bicistronic RNA or GFP alone. Transfected cells were stained to detect nuclei (DAPI) and F-actin (rhodamine phalloidin). Digitally cropped and magnified insets showing syncytia from the AvRVG images are shown to the right of the GFP image.

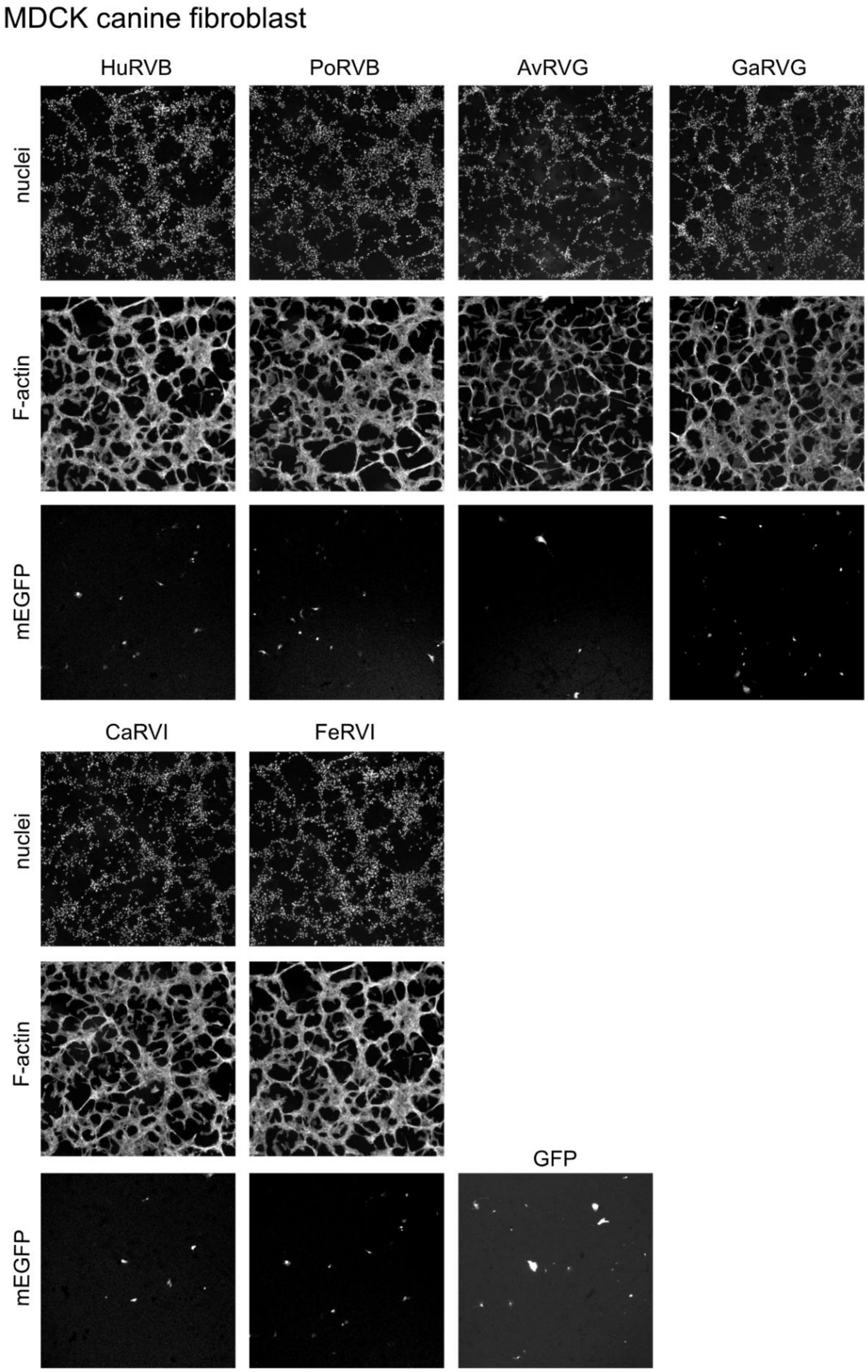

Figure S8. Detection of nuclei, actin, and mEGFP in MDCK canine fibroblasts transfected with plasmids expressing a bicistronic RNA. MDCK cells were transfected with plasmids encoding RVB, RVG, or RVI NSP1-1 and mEGFP separated by an IRES on a bicistronic RNA or GFP alone. Transfected cells were stained to detect nuclei (DAPI) and F-actin (rhodamine phalloidin).
